# *P21* ^cip^-Overexpression in the Mouse β Cells Leads to the Improved Recovery from Streptozotocin-Induced Diabetes

**DOI:** 10.1371/journal.pone.0008344

**Published:** 2009-12-17

**Authors:** Jie Yang, Weiqi Zhang, Wei Jiang, Xiaoning Sun, Yuhua Han, Mingxiao Ding, Yan Shi, Hongkui Deng

**Affiliations:** 1 The MOE Key Laboratory of Cell Proliferation and Differentiation, College of Life Sciences, Peking University, Beijing, China; 2 Laboratory of Chemical Genomics, Shenzhen Graduate School of Peking University, Shenzhen, China; 3 Beijing Vitalstar Biotech Co., Ltd., Beijing, China; 4 Beijing Laboratory Animal Research Center, Beijing, China; University of Bremen, Germany

## Abstract

Under normal conditions, the regeneration of mouse β cells is mainly dependent on their own duplication. Although there is evidence that pancreatic progenitor cells exist around duct, whether non-β cells in the islet could also potentially contribute to β cell regeneration in vivo is still controversial. Here, we developed a novel transgenic mouse model to study the pancreatic β cell regeneration, which could specifically inhibit β cell proliferation by overexpressing *p21* ^cip^ in β cells via regulation of the Tet-on system. We discovered that *p21* overexpression could inhibit β-cell duplication in the transgenic mice and these mice would gradually suffer from hyperglycemia. Importantly, the recovery efficiency of the *p21*-overexpressing mice from streptozotocin-induced diabetes was significantly higher than control mice, which is embodied by better physiological quality and earlier emergence of insulin expressing cells. Furthermore, in the islets of these streptozotocin-treated transgenic mice, we found a large population of proliferating cells which expressed pancreatic duodenal homeobox 1 (PDX1) but not markers of terminally differentiated cells. Transcription factors characteristic of early pancreatic development, such as *Nkx2.2* and *NeuroD1*, and pancreatic progenitor markers, such as Ngn3 and c-Met, could also be detected in these islets. Thus, our work showed for the first time that when β cell self-duplication is repressed by *p21* overexpression, the markers for embryonic pancreatic progenitor cells could be detected in islets, which might contribute to the recovery of these transgenic mice from streptozotocin-induced diabetes. These discoveries could be important for exploring new diabetes therapies that directly promote the regeneration of pancreatic progenitors to differentiate into islet β cells in vivo.

## Introduction

For both type 1 and type 2 diabetes mellitus, one of the most critical pathogeneses is that the number of functional β cells is inadequate [1]. Transplantation with donor islets has succeeded in diabetic clinical trials; however, the resources for transplantable islets are limited [2], [3]. Beside islet transplantation therapy, another potential clinical approach is to stimulate endogenous β-cell regeneration in diabetic patients [4]. Hence, it is important to understand the mechanisms that regulate β-cell regeneration in the islets of diabetic patients.

To study the mechanisms underlying β-cell regeneration, several experimental models have been developed, including the chemical induction of diabetes [5], partial pancreatectomy [6], duct ligation or cellophane wrapping [7] and abnormal expression of destructive genes such as *TGFα* [8], diphtheria toxin A [9] and c-Myc [10]. With these models, previous studies have demonstrated that the maintenance and regeneration of β cells relies mainly on the proliferation of terminally differentiated β cells [11]. Further results from label retention analysis also indicate that all β cells are equal in replication capacity [12]. On the other hand, it has also been recently demonstrated with a unique mouse model of pancreatic damage (i.e., partial duct ligation), that the NGN3+ cells in duct lining could reappear in the adult pancreas following injury and differentiate into new β cells in transplanted mice [13]. Although these two mechanisms (i.e., β-cell self-replication and reactivation of pancreatic progenitors) for β-cell regeneration have been demonstrated, there are still no direct evidences to indicate whether there are progenitor cells in islets, which could be re-activated for β-cell regeneration.

Here, we develop a novel mouse model that could specifically inhibit β-cell replication by the inducible expression of *p21* in the β cells of adult mice. With this model, we show the evidence for the first time that overexpression of *p21* in mice islets could improve the recovery from streptozotocin-induced diabetes.

## Results

### Doxycycline-Inducible Regulation of *p21* Overexpression in Islet β Cells

**Figure 1:**
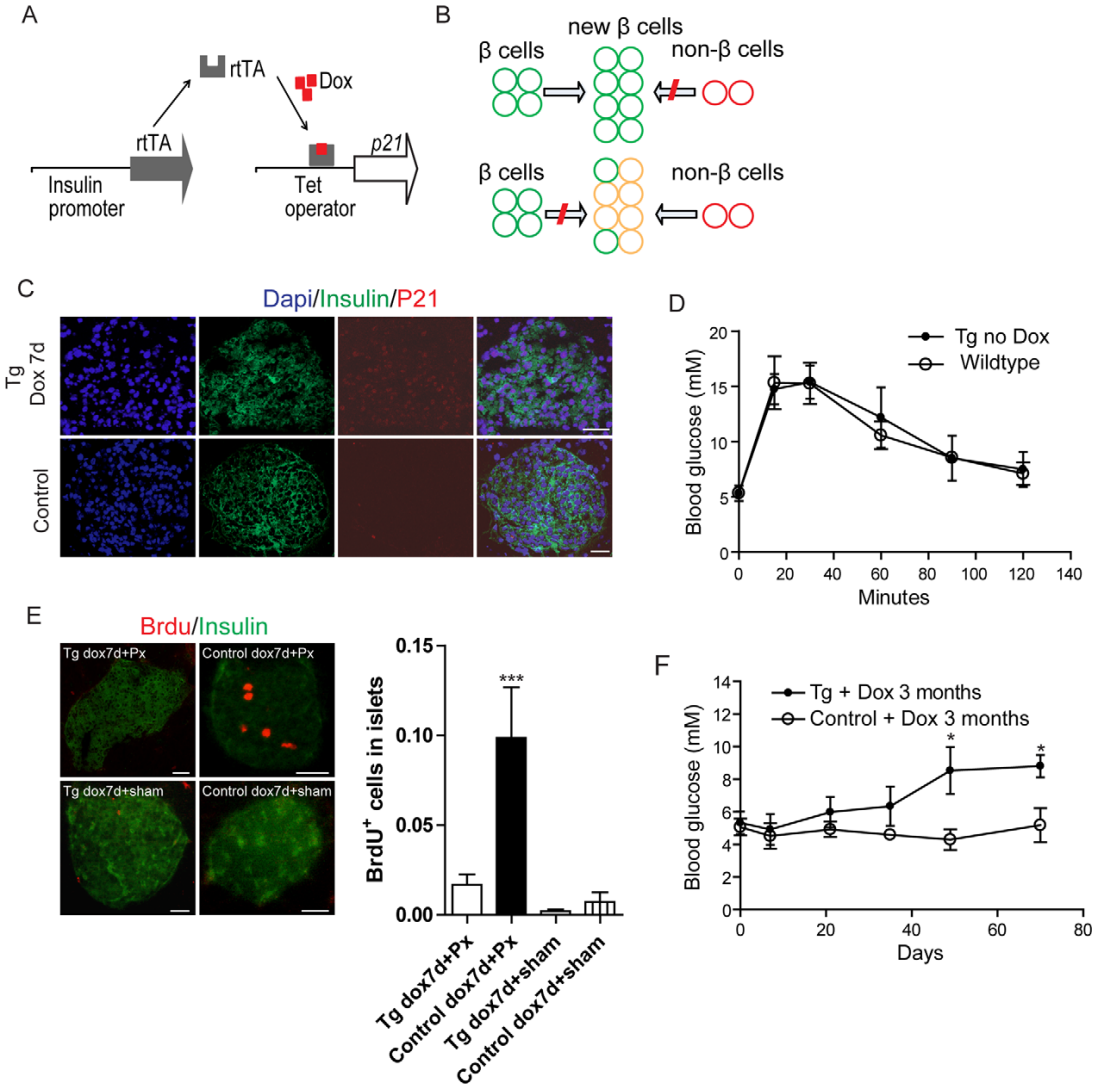
Generation of transgenic mice conditionally over-expressing *p21* in islets. (A) Schematic of the Tet-on regulated system. Addition of doxycycline (Dox) to the drinking water of Insulin-rtTA; the TET-*p21* double transgenic mice could induce the over-expression of *p21* specifically in β cells. (B) Experimental design: Top, under normal conditions, the regeneration of β cells mainly depends on self-replication; below, when repress the self-duplication of β cells, non-β cells might be re-activated and contribute to the regeneration. (C) Tissue-specific expression of *p21* in islet β cells. Six-week old TET-*p21* double-transgenic mice or control mice were treated with dox for 7 days and check the expression pattern of *p21*. Specific over-expression of *p21* in Insulin-expressing cells was confirmed by immunofluorescence staining for *p21* (red) and Insulin (green) in TET-*p21* double-transgenic mice. Scale bar: 40 µm. (D) In the absence of dox, 5-week-old TET-*p21* double-transgenic mice had normal fasting blood glucose levels and glucose tolerance. Values are mean±SEM (*n* = 6). (E) Left, TET-*p21* transgenic Px mice showed fewer BrdU-positive β cells than control Px mice after 7 days of dox administration, assessed by immunofluorescence staining for Insulin (red) and BrdU (green). Sham-operated mice were used as negative control, the difference between transgenic Px mice and transgenic sham-operated mice is not statistical significant. Right, Quantification of the BrdU labeling β cells in double-transgenic Px mice treated with Dox for 7 days is much lower than the cells in control mice. Data are shown as percentage±SEM of BrdU-labeled cells contributing to the total β-cell population. ***P<0.001, n≥8. Scale bar: 50 µm. (F) Blood glucose change during 3 months of dox administration. Six week old transgenic mice and their littermates were treated with Dox for more than 3 months, during this period, the blood glucose levels were detected every 7 days, the blood levels of transgenic mice were clearly higher than control littermates. *P<0.05, n≥5. Tg: double transgenic mice, Control: Single-transgenic TET or *p21* littermates.

To study whether non-β cells in islets could be activated and contributed to β cell regeneration in some specific pathologic conditions such as β cell loss, we generated a double transgenic mouse model with the Tet-On system (Insulin-rtTA/TET-*p21*); the foreign gene *p21* is controlled by the RIPII-rtTA promoter. Therefore, in this transgenic mouse model, doxycycline (dox) treatment induces the specific overexpression of *p21* in islet β cells (Fig. 1A), which can inhibit the proliferation of β cells, thus facilitating our study of the mechanism on β-cell regeneration in islets with pancreatic progenitor or precursor activation (Fig. 1B). The transgenic mice developed normally without doxycycline treatment. Exposed to doxycycline for 7 days, these transgenic mice could be shown to be specifically expressing *p21* in adult islet β cells using immunofluorescence analysis of pancreatic tissue (Fig. 1C). There was no obvious difference in glucose homeostasis between Insulin-rtTA/TET-*p21* double-transgenic mice and the wild-type mice (Fig. 1D). Furthermore, although *p21* is sometimes involved in apoptosis, the double transgenic mice treated with doxycycline for 7 days did not exhibit abnormal level of apoptosis in islet (Fig. S1A).

### *P21* Overexpression in β Cells Inhibits β-Cell Proliferation

The rate of pancreatic β-cell replication is extremely slow [14] (Fig. 1E), but β cells can be coaxed to replicate more quickly by a variety of maneuvers and physiological stimuli, including subtotal pancreatectomy [11], in which β-cell regeneration mainly depends on β-cell self-duplication. Therefore, to explore the question that whether *p21* expression in β cells is sufficient to inhibit their cell cycle progress, we first induced *p21* expression in adult islets in double transgenic mice with dox treatment, and then performed a 70% partial pancreatectomy (Px) or sham-operation on transgenic and control mice. The pancreatic remnants (which correspond to the duodenal pancreas) were analyzed two days after the operation. BrdU incorporation was utilized to detect the β-cell replication. We discovered that the replication of β cells was dramatically decreased in Px transgenic mice overexpressing *p21* compared with Px normal mice (single transgenic mice) (Fig. 1E). Quantification of proliferation percentage using BrdU staining revealed that β-cell proliferation rates were 5.7±1.7% in the Px control group versus 1.7±1.0% in the Px *p21* transgenic groups (Fig. 1E). These data indicated that β-cell proliferation was limited by *p21* overexpression after pancreatectomy.

To further elucidate whether p21 could repress β-cell proliferation, we traced the serum glucose levels of *p21* transgenic mice for a long term. Single transgenic littermates were used as control. After dox administration for 3 months to continuously up-regulate *p21* expression in β cells, these mice gradually suffered from hyperglycemia (Fig. 1F). TUNEL staining showed that β cells undergone apoptosis in these transgenic mice (Fig. S1B). This result also supported that β-cell homeostasis mainly depends on the maintenance of β cell mass as previously reported [9], [11], [15], [16].

### *P21* Overexpression Improved the Recovery from Streptozotocin (STZ) -Induced Diabetes

**Figure 2:**
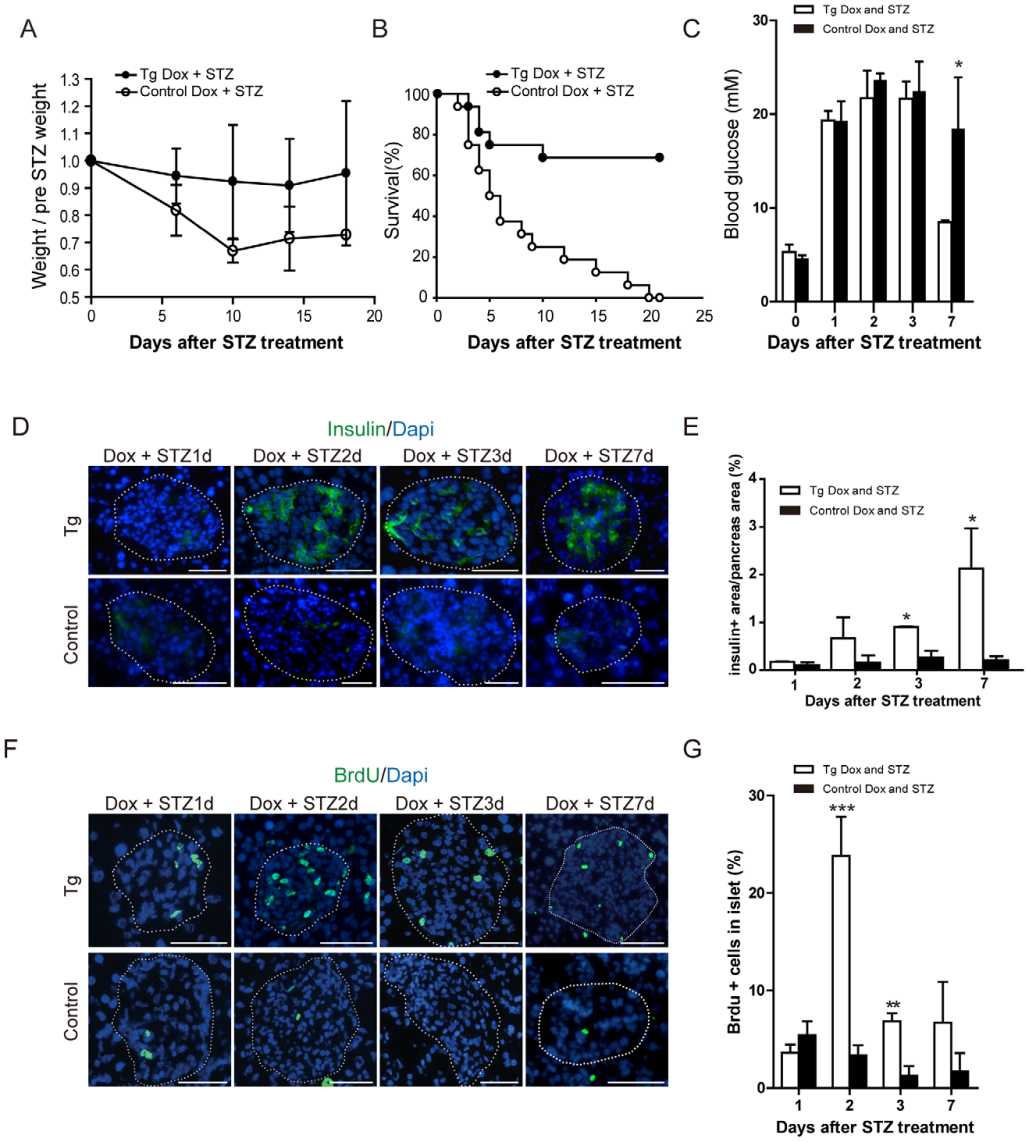
*P21*-overexpresing improved the recovery from STZ-induced diabetes. Following administration of dox for one week, double transgenic mice and their littermates were treated with 200 mg/kg of STZ and (A) weight, (B) diabetes-specific survival, (C) serum glucose levels were tested up to 20 days after STZ treatment. Mice were administrated with dox during this experimental process. *P<0.05, n≥11. (D) Pancreas tissue from double transgenic and control mice were collected on day 1, day 2, day 3, day 7 after STZ treatment, assessed by immunofluorescence staining for insulin (green). Morphometric analysis revealed β-cell destruction one day after STZ treatment (STZ 1d) in *p21*-overexpressing transgenic mice and control mice; extensive Insulin-positive β regeneration could be detected in the islet of double transgenic mice two days after STZ treatment (STZ 2d, STZ3d, STZ 7d) and no Insulin-positive cells could be detected in control mice. Scale bar: 20 µm. (E) Quantifications of β-cell regeneration based on insulin staining and intact cellular morphology were detected 1, 2, 3, 7 days after the STZ injection and ceaseless dox treatment. *P<0.05, n≥3. (F) Islet cell proliferation after STZ treatment in *p21*-overexpressing mice and control mice measured by BrdU (green) labeling. In the islets of double transgenic mice, BrdU incorporated cells emerge one day after STZ treatment, increase dramatically in two days after STZ treatment, and then decline afterward; in control mice, BrdU incorporated cells are very few all the time. (G) The number of BrdU+ cell in islets on pancreatic tissue sections was about 5-fold higher in the double transgenic mice versus control pancreas. ***P<0.001, **P<0.01, n≥6. Islets were indicated by dashed circle. Tg: *p21*-overexpressing transgenic mice, Control: Single-transgenic TET or *p21* littermates.

To further study whether non-β cells contribute to β-cell regeneration when most β cells were destroyed and failed to duplicate themselves, we adopted a model of islet injury by streptozotocin treatment. As a specific β-cell toxin, streptozotocin can induce β-cell necrosis and diabetes when given as a single dose, and limited β-cell regeneration after STZ treatment has been reported [17], [18]. Double transgenic mice and control mice (single transgenic littermate) were supplemented with dox for 8 days, 9 days, 10 days or 14 days respectively, during our experiments to initiate and maintain *P21* expression. At the eighth day of dox administration, mice were first treated with a high dose of STZ (200 mg kg^−1^), and then their weight (Fig. 2A), diabetes-specific survival capability (Fig. 2B), and serum glucose levels (Fig. 2C) were measured. Surprisingly, we discovered that the *p21*-overexpressing transgenic mice obviously promoted the recovery from STZ injury (Fig. 2). After the same dose of STZ treatment, *p21*-overexpressing mice were far more competent to increase weight (Fig. 2A), survive (Fig. 2B), and regain normoglycemia (Fig. 2C) than normal littermates.

We also tried to determine the amount of β-cell regeneration after STZ treatment. One day after STZ injection, almost all β cells in islets were eliminated. Sections of pancreas stained with Insulin revealed that the islets of both *p21*-overexpressing transgenic and control mice contained cell debris and scattered nuclei, instead of stained living cells (Fig. 2D, STZ 1d), as previously reported [19], [20]. Two days after STZ treatment, Islets of control group also lacked β cells and still contained cellular debris; in contrast to the control mice, most of the *p21*-overexpressing islets contained a number of newborn Insulin-positive cells two days after STZ treatment (Fig. 2D, STZ 2d). Seven days after STZ treatment, the average percentage of insulin positive cell in transgenic islets could restored to 27.1±3.9%, while in control mice the percentage only arrived 5.7±2.9%. Quantification of β cell area in pancreas also revealed that β cells regeneration in *p21*-overexpressing transgenic mice. From the first day to the seventh day after STZ treatment, the percentage of β cell area of total pancreas area in transgenic mice was 0.17±0.01% (one day), 0.58±0.56% (two day), 0.91±0.01% (three day) and 2.42±1.13% (seven day) of the islet mass; while the percentage of control mice was 0.11±0.06% (one day), 0.16±0.19% (two day), 0.26±0.14% (three day) and 0.21±0.08% (seven day) of the islet mass (Fig. 2E).

Because the self-duplication of β cells was inhibited in transgenic mice, this recovery from STZ treatment and emergence of newborn Insulin-positive cells suggested that mechanisms other than β-cell self-renewal contributed to β-cell regeneration when β cells were destroyed.

### A Large Population of Proliferating Cells Expressing pdx1 Emerges in the Islets of *p21*-Overexpressing Mice after Injury

**Figure 3:**
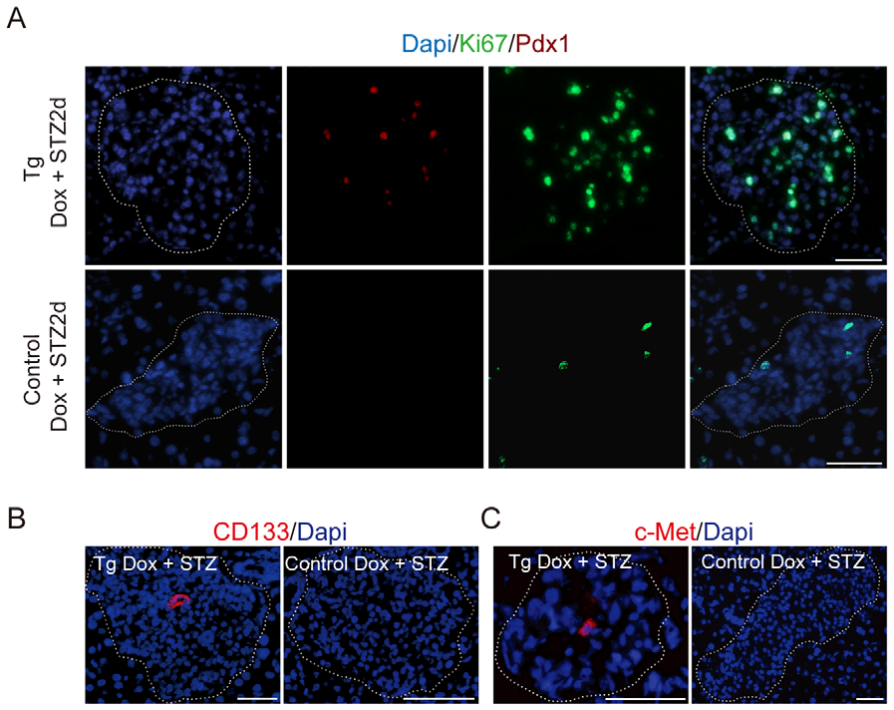
Properties of the proliferating cells in *p21*-overexpressing islets. In addition to persistent administration of dox, double transgenic mice and their littermates were treated with 200 mg/kg of STZ after *p21* over-expression was induced. (A) Two days after STZ treatment, PDX1+ cells were almost eliminated in the islet of control mice due to β-cell loss; while PDX1+ (red) BrdU+ (green) cells were emerged in the islet of double transgenic mice. Scale bar: 20 µm. (B) CD133 (red) was detected in islets of *p21*-overexpressing mice by immunofluorescence but not in control mice. *P21* Tg: *p21*-overexpressing transgenic mice, Control: Single-transgenic TET or *p21* littermates. (C) c-Met (red) was detected in islets of p21-overexpressing mice by immunofluorescence while no c-Met was expressed in control mice. Scale bar, 50 µm. Islets were indicated by dashed circle. Tg: *p21*-overexpressing transgenic mice, Control: Single-transgenic TET or *p21* littermates.

To define which cells in the islets proliferate and contribute to β-cell regeneration, we detected proliferating cells in the islets of *p21*-overexpressing transgenic mice following STZ injection. Using BrdU incorporation, we found a large group of proliferating cells in the islets emerged one day after STZ treatment, increasing to a peak on the second day, and then declining (Fig. 2E–F). Two days after STZ treatment, about 25% cells in islets of *p21* overexpressing transgenic mice were proliferating; while in control mice the percentages were all less than 4% (Fig. 2E–F). Double-labeling with BrdU and the endocrine markers including Insulin, Glucagon, Somatostatin, and pancreatic polypeptide demonstrated that these proliferating cells were not endocrine cells (Fig. S2, S3), which excluded the possibility that the burst of unblocked β-cell replication might happen in transgenic mice with dox treatment. We also checked the markers for other cell types that exist in islets, such as βIII-Tubulin, GFAP, vimentin and CD45, but none of the proliferating cells expressed these markers (Fig. S2B–C). In order to identify these proliferating cells(i.e. Ki67 positive), we screened some early transcription factors and putative progenitor markers in pancreas, and found out about 22% proliferating cells expressed Pdx-1. However, in the control mice, we did not find any PDX1+ proliferating cells (Fig. 3A).

Some early transcription factors and putative progenitor markers are also expressed in the islets of *p21*-overexpressing transgenic mice after STZ induced islet injury (Fig. 3B–C). It has been demonstrated that CD133 and c-Met are pancreatic progenitor/stem cell markers [21], [22]. In the islets of adult *p21*-overexpressing transgenic mice, we detected the expression of CD133 and c-Met two days after STZ injection. On the contrary, although expression of CD133 was detected in some duct-like structures outside of the islets, no CD133- or c-Met-positive cells could be discovered in the adult islets of single transgenic littermates after STZ injection (Fig. 3B–C).

**Figure 4:**
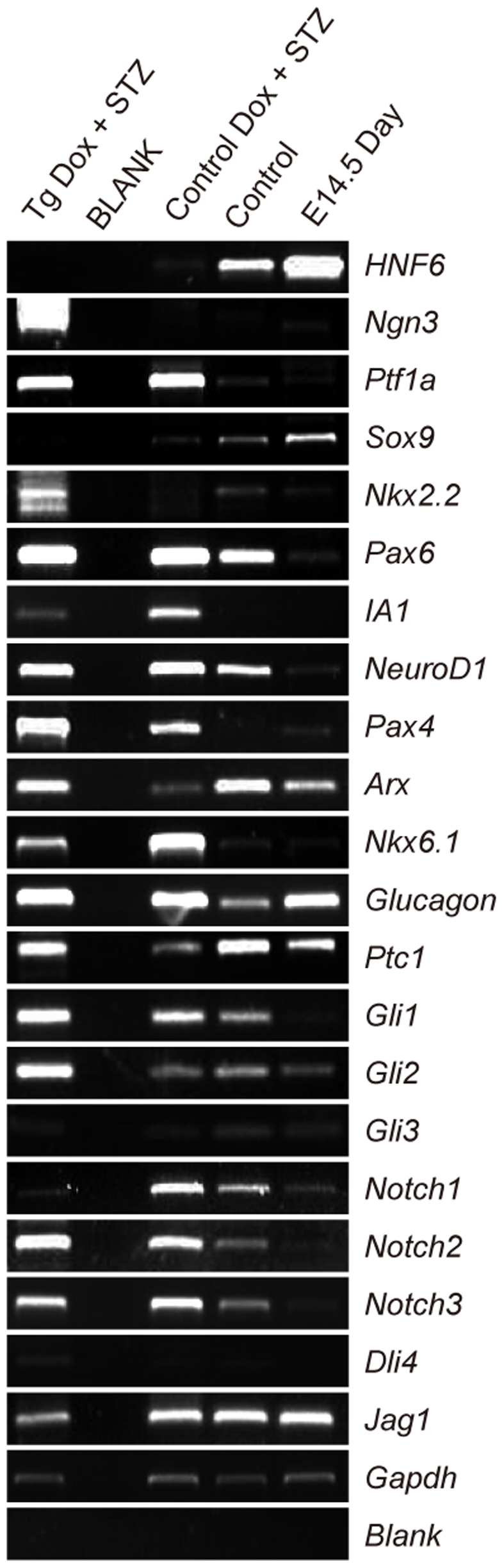
RT-PCR analysis of islets. Six-week-old mice were treated with dox for 7 days, and then injected with 200 mg/kg STZ. Two days after STZ treatment, islets are isolated to perform RT-PCR analysis. Compared to islets of control mice, RT-PCR could detect the expression of progenitor marker *ngn3* and pancreatic transcription factors *Pax4*, *Arx*, *Nkx2.2*, *NeuroD1*, *Pax6*, which regulated by *Ngn3*, was high in double transgenic islets two days after STZ treatment. cDNA from adult mouse islet cells and from E14.5 pancreas served as control. The negative control (Blank) contained water without cDNA.

More extensive gene-expression profiling showed that Ngn3 and the transcription factors downstream of *Ngn3 (Pax4, Arx, Nkx2.2, NeuroD1* and *Pax6*) were also activated in the injured islets of transgenic mice (Fig. 4). A more than 300-fold increase of Ngn3 expression in transgenic islets was confirmed by Real-time PCR, which was more than three times higher than that of control mice after STZ treatment as previously reported [23] (Fig. S4). These results suggested that when treated with STZ, the islet progenitor cells of *p21*-overexpressing transgenic mice might be reactivated.

### Cells Co-Expressing Two or Three Kinds of Endocrine Hormones Are Detected in *p21*-Overexpressing Transgenic Mice after Injury

**Figure 5:**
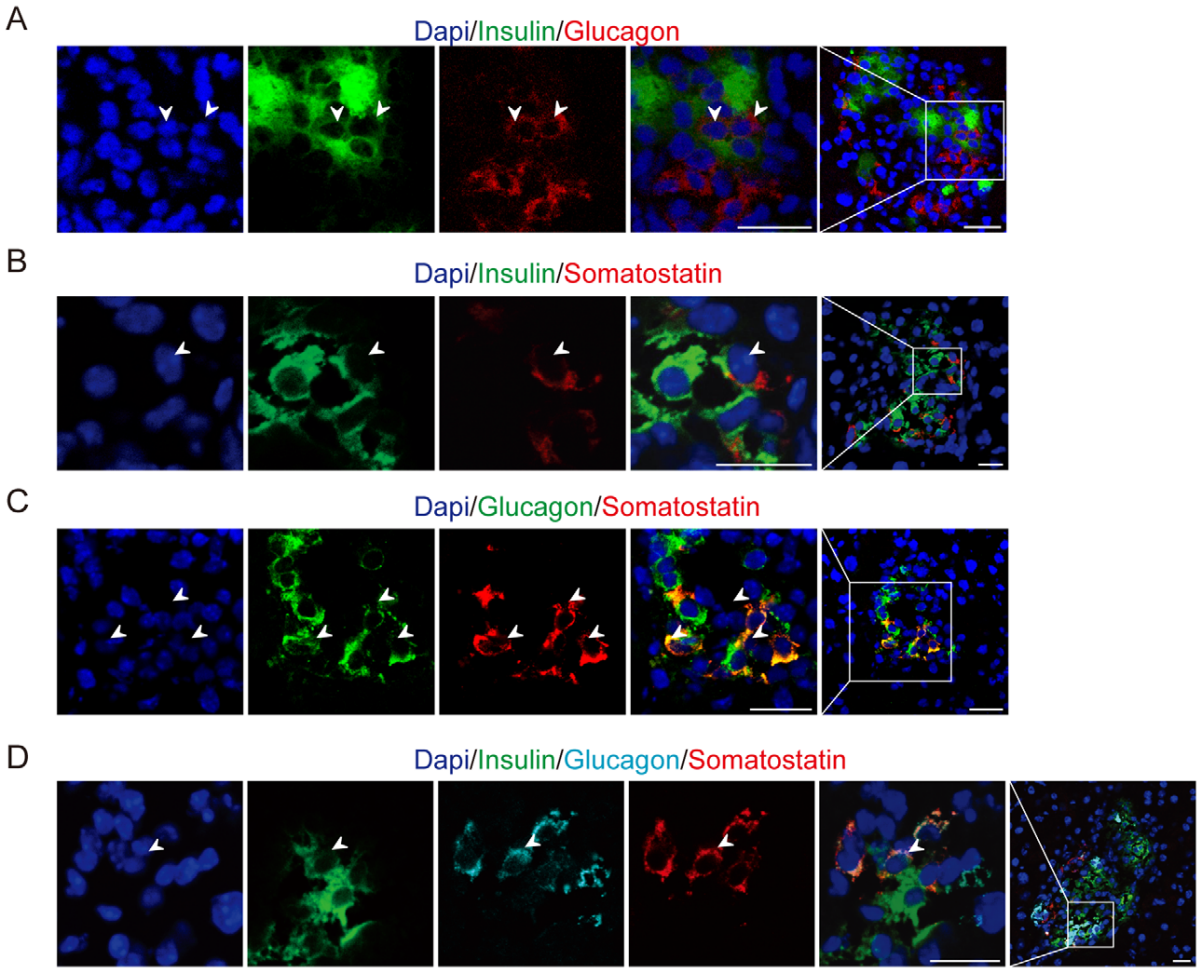
Immature progenitor state of the islet cells in *p21*-overexpressing transgenic mice after STZ treatment. (A–C) Representative confocal images of co-staining with two islet endocrine markers in *p21*-overexpressing transgenic mice after STZ treatment. Dapi, Blue. (A) The islet cells co-expressing Insulin (green) and Glucagon (red). (B) The islet cells co-expressing Insulin (green) and Somatostatin (red). (C) The islet cells co-expressing Glucagon (red) and somatostatin (green). Scale bar: 20 µm. (D) Representative confocal images of Insulin and Glucagon and Somatostatin co-staining in STZ-injured double transgenic mice. Pancreas sections were stained for dapi (blue), Insulin (green), Glucagon (cyan) and Somatostatin (red). Arrowhead denotes a cell co-expressing three or two endocrine markers. Scale bar: 16 µm.

To further investigate the mechanisms that contribute to STZ resistance in our mouse model, we then studied the structural changes in islets. The proportions and locations of endocrine hormones such as Insulin, Glucagon, Somatostatin and pancreatic polypeptide were checked. After STZ treatment, the proportion of Glucagon-expressing and Somatostatin-expressing cells increased, and the morphology of *p21*-overexpressing islets after injury was disturbed, in that Insulin-expressing cells were not spatially surrounded with Glucagon-expressing cells as in normal islet (data not shown). Endocrine hormone co-expression was detected in this injury model. One day after STZ treatment, some cells in *p21*-overexpressing islets expressed Glucagon and Somatostatin simultaneously. Two days after STZ treatment, 72.8±4.9% of the Glucagon-expressing cells also expressed Somatostatin, and some cells simultaneously expressed Insulin and Glucagon, or Insulin and Somatostatin (Fig. 5A–C). A small group of cells (5.4±1.4%) even produced Insulin, Glucagon and Somatostatin (Fig. 5D), whereas in single transgenic mice after STZ, no such cell were detected (Fig. S5). This group of cells produced two or three endocrine hormones were also found in mice constitutively overexpressing *p21*. However, the number of these immature cells detected in the STZ-treated *p21*-overexpressing transgenic mice was much higher than that in the untreated transgenic mice (data not shown), which suggesting that islet hormone co-expressing cells were more strongly activated following islet acute injury.

## Discussion

Our study developed a novel transgenic mouse model to explore whether non-β cells in islets could contribute to β-cell regeneration when β-cell self-replication was repressed. This transgenic mouse model could inducibly overexpress *p21* under the control of the Tet-on system specifically in islet β cells, which could inhibit the replication of β cells directly in vivo. *P21* is an inhibitor of the cell cycle [24], [25] and *p21* absence in islets had no effects on islet mass and glucose metabolism [24]. However, we first detected that continuously overexpressing *p21* over time slowly elevated the level of serum glucose and eventually caused diabetes (Fig. 1F). And our data demonstrated that overexpression of *p21* in the mouse pancreas efficiently arrested cell cycle and progressively caused apoptosis (Fig. 1E–F, Fig. S1), similar to its effect in hepatocytes and renal cells [26], [27]. Therefore, our transgenic mouse model was demonstrated to be valuable for the study of non β-cell derived β-cell regeneration in islet.

Moreover, we also discovered that this transgenic mouse model could enhance recovery against STZ-induced diabetes, with lower blood glucose levels after STZ injury, higher weight and survival efficiency (Fig. 2A–C). The Insulin-producing cells in the islets of these transgenic mice could reappear two days after STZ treatment (Fig. 2D). These phenomena suggest that when β-cell self-duplication is repressed, islet damage by STZ treatment might re-activate other mechanisms to induce β-cell regeneration in our mouse model for diabetic recovery.

We detected a large population of proliferating cells in *p21*-overexpressing islets of our mouse model following STZ injection, which did not express any terminally differentiated islet cell markers (Fig. S2A) including Insulin (Fig. S3), or the markers of other differentiated cells, such as neurons (βIII-Tubulin), glia cells (GFAP), vimentin and blood lineages (CD45) (Fig. S2B–C). The evidences that these proliferating cells could undergo significant replication and expressed no differentiated markers suggests that these cells might be “transient amplifying cells” in the process of “stem cells→ transient amplifying cells→ terminally differentiated cells” [28]. Moreover, we demonstrated that several transcription factors critical for pancreatic progenitor development were also up-regulated in the islets of our P21 overexpression transgenic mice after STZ treatment (Fig. 3B–C, Fig. 4, Fig. S4). One of these factors Pdx1 is the key transcription factor which regulates the embryonic development of pancreas and expresses widely in the embryonic pancreatic progenitors [29]. PDX1+Ki67+ cells could be activated in 60% Px model around duct and after in vitro culture of β cells [30], [31]. Kodama and Araki group also reported that pdx1 and ngn3 would be activated after STZ treatment, which was related to beta cell regeneration [23]. Ngn3 is the earliest and specific islet progenitor marker during embryonic development but do not express in adult pancreas [32]. Therefore, we presumed that re-expression of ngn3 and other embryonic pancreatic progenitor markers such as Pax4, Arx, and Nkx2.2 after STZ induced islet injury might indicate the islet progenitor reactivation in the P21 transgenic mice (Fig. 4). In our P21 transgenic mouse model, Pdx1 could be detected in about 22% proliferating cells in the islet of STZ treated transgenic mice (Fig. 3A), while Ngn3 was up-regulated more than 300 times in p21 overexpressing mice after STZ treatment than that of control mice. Ngn3 was considered to be an unambiguous marker known for islet progenitors in embryonic [32] and in the adult pancreas [13], which. Previous studies also showed that c-Met could be detected around the acini and ducts in normal philosophy, and CD133 used to isolate pancreatic NGN3+ cells from fetal mice and humans could be potential pancreatic stem/progenitor cell markers [21], [22]; The expression of these two genes were also found in the islets of our mouse model after injury. However, none of these genes expression was detected in the islets of control mice following STZ injury. Altogether, our results indicated that the putative progenitor cell activation could be induced under conditions where β-cell self-duplication is inhibited.

On the other hand, in the islets of *p21*-overexpressing transgenic mice following STZ treatment, we clearly identified simultaneously co-expression of two or more endocrine markers such as Insulin, Glucagon and Somatostatin (Fig. 5A–D). It has been demonstrated that during early pancreas development, there are cells co-expressing Insulin, Glucagon, and Somatostatin spontaneously, which are multipotent islet precursors and can differentiate into all endocrine cell types [33], [34]. Therefore, our data suggested that β-cell regeneration in the adult islets might be the reason of the STZ-induced diabetic recovery in P21 transgenic mice, which might involve the activation of early pancreatic developmental pathways.

In summary, our work established a novel transgenic mouse model to uncover the detailed mechanisms of β-cell regeneration in islets, which could have critical potential for future diabetes therapy.

## Materials and Methods

### Generation of Transgenic Mice

All experiments were approved by the Animal Care and Use Committee of Peking University. Our Transgenic mouse model were generated on a pure CD1 background by cross breeding two transgenic lines of mice: one carrying the p21 regulated by a bidirectional tetracycline responsive element and the second expressing the reverse tetracycline transactivator (rtTA) under the control of the rat insulin II promoter Transmission of both transgenes was monitored by PCR analysis from tail DNA by using the following primers:

1. TCAAAAGCTGGGAGTTGAGC
2. CTCAGAAGTGGGGGCATAGA
3. GGATCCTCTAGTCAGCTGAC
4. GAGGAAGTACTGGGCCTCTT

The transgenes were activated in vivo by administrating 1 mg/ml of dox in 1% sucrose in the drinking water (the control mice received only 1% sucrose). Water was changed every 5 days. 6-week old mice were treated with dox for 7 days to initiate the expression of *p21*, and then sacrificed to check for the pattern of *p21* expression. Three independent transgenic mouse lines with right pattern were used for following studies. Single transgenic mice were used as control mice.

### Determination of Apoptosis

To assess the apoptosis by TUNEL assay, dissected pancreas were first fixed with 4% paraformaldehyde and then permeabilized with 0.5% TritonX-100. The TUNEL procedure was performed under the instruction of the DeadEnd fluorometric TUNEL system (Promega) and then viewed using the normal Leica microscopy. Positive control cover slips were treated with DNase I (Promega) prior to the addition of the 3′-OH labeling mix according to the instruction. All samples were analyzed with at least two biological replicates, and three images from each replicate.

### Pancreatectomy and STZ Treatment

Double transgenic male mice and control mice (single transgenic male mice) aged 6 weeks were administrated with dox in the whole process of our experiments. After one week of dox administration to initiate p21 expression, we performed Px and STZ injection. For the Px model, these two groups (at least 5 mice per group) underwent partial (60%) Px and were sham operated. For the single high-dose STZ model, these two groups (at least 20 mice per group) were injected i.p. with STZ (Sigma) dissolved in citrate buffer, pH 4.5, at a concentration of 200 mg/kg.

To measure the islet proliferation after treatment, BrdU (Sigma-Aldrich, 200 mg/kg) was administered 6 h before sacrifice. BrdU incorporation was assessed using a BrdU-specific mouse antibody (Zymed). Then BrdU-positive cells and total cell numbers of islet in the whole randomly selected microscopic fields were counted from three non-consecutive slides of each mouse. The percentage of BrdU-positive cells was calculated by the number of BrdU-positive cells/the total number of islet cells.

### Glucose Level Determinations

After 3 months of dox administration or STZ injection, Glucose levels (mmol/l) of mice were determined with the Accu-Chek Active meter (ROCHE) with blood sample obtained from the snipped tail after overnight fast. For intraperitoneal glucose tolerance test, mice were fasted overnight (16–18 h), then their fasting basal glucose was measured, and they were injected intraperitoneally with 25% glucose in saline to a final amount of 2 g glucose/kg body weight. Blood samples were obtained from the snipped tail at 15, 30, 60, 90, and 120 min after glucose injection and analyzed.

### Immunofluorescence

Mice were killed by cervical dislocation, and then adult tissues were dissected. Tissues were embedded, frozen in tissue-Tek OCT compound, and serially sectioned. At least three nonconsecutive individual sections per animal were selected for each immunostaining analysis. For immunoflurescence staining, frozen sections were rehydrated and washed in PBS, permeabilized with 0.1% Triton X-100 in PBS (15 minutes, room temperature), and incubated with primary and secondary antibody (TRITC or fluorescein or CY5-conjugated). Stained sections were mounted (Vector) and analyzed on a Leica confocal microscope. The following primary antibodies were used: guinea pig anti-Insulin (1∶200; DAKO), rabbit anti-Glucagon (1∶200; Santa-cruz), goat anti-Somatostatin (1∶200; Santa-cruz), rabbit anti-pancreatic polypeptide (1∶200; Chemicon), mouse anti-BrdU (1∶80; Zymed), mouse anti-ki67 (1∶100; DakoCytomation), Rabbit anti-pdx1 (1∶200; Abcam), rabbit anti–c-Met (1∶150; Santa-cruz), and rabbit anti-CD133 (1∶200; Santa-cruz), rabbit anti-P21 (1∶100; Santa-cruz), mouse anti-Vimentin (1∶200; Santa-cruz), rabbit anti-CD45(1∶200; BD), rabbit anti-βIII-Tubulin (1∶200; Santa-cruz), rabbit anti-GFAP (1∶200; Santa-cruz). Secondary antibodies were all from Jackson Immunoresearch Laboratories. For double and triple staining, only affinity-purified secondary antibodies were used. Images were viewed using Microscope (Leica DM4000B) or confocal scanning (Leica DMIRE) microscopy.

### Histological Analysis

Quantification evaluation of β-cell area was performed after insulin staining of pancreas. To measure beta cell mass, three randomly selected insulin stained pancreas sections from each mouse (n = 3) were imaged. Pancreatic and islet areas were outlined, quantified and are presented as a ratio as reported previously by Dr. Cano [35]. To measure the average percentage of insulin positive cells in islet, more than 60 islets of each mouse (n = 3) were analyzed, calculating the ratio between the area occupied by insulin-positive cells with intact cellular morphology and that occupied by the islets.

### RT-PCR and Real-Time PCR Analysis

Mouse islets were isolated as described previously [36]. In the double transgenic mice and control mice two days after STZ treatment, pancreas were injected through the pancreatic duct with 3 ml of 1.7 mg/ml Collagenase P (Gibco) in Hanks' buffered saline solution (HBSS), removed, incubated at 37°C for 17 min, and then passed through a 500-µm wire mesh. The digested pancreas was rinsed with HBSS, and islets were separated by density gradient in Histopaque (Sigma). After several washes with HBSS, islets were handpicked under a microscope. After isolation, islets were aliquoted and stored at −70°C until RNA was isolated. Semiquantitative RT-PCR was performed using the primer pairs shown in Table S1. Real-time PCR analysis was performed on ABI PRISM 7300 Sequence Detection System using the SYBR Green PCR Master Mix (TOYOBO) by Ngn3 specific primes.

### Statistics

All data presented are representative of at least three independent experiments unless indicated otherwise. The results are expressed as the mean±SEM of at least three independent experiments. Statistical analysis was performed using one-way ANOVA.

In all of the tests, value of *P*<0.05 was considered significant, *P*<0.01 highly significant, and *P*<0.001extremely significant.

## Supporting Information

Figure S1 *P21* over-expression caused apoptosis in double transgenic mice after dox treatment up to 3 months. Six-week-old mice were treated with doxycycline for one week (A) and 3 months (B), then sacrificed and assessed for apoptotic cell death. On 7th day, little β cell apoptosis was detected by TUNEL staining in double-transgenic mice as that in wildtype mice. After 3 months, β cell apoptosis was detected only in transgenic mice. Pancreatic slides treated with DNase I prior to the addition of the 3′-OH labeling mixture were utilized as positive control for this experiment. Islets were indicated by dashed circle. Scale bar: 50 µm.
(2.21 MB TIF)

Figure S2 Proliferating cell did not co-stain with markers of terminally differentiated cells in islet. (A) Double-labeling with BrdU (red) and endocrine markers (green) such as Insulin, Glucagon, Somatostatin, and pancreatic polypeptide demonstrated that these proliferating cells are not endocrine cells - α, β, δ or pancreatic polypeptide cells. (B) Staining with Ki67 (green) and βIII-tubulin and GFAP (red) revealed that these proliferating cells are not neurons or astrocytes. (C) Staining with BrdU (red) and vimentin and CD45 revealed that these proliferating cells are not hematopoietic cells or mesenchymal cells. Scale bar, 50 µm.
(0.79 MB TIF)

Figure S3 Proliferating cell did not co-stain with insulin in islet. Six-week-old mice were treated with dox for 7 days, and then injected with 200 mg/kg STZ. Two days after STZ treatment, Double-labeling with BrdU (red) and insulin (green) was checked by confocal microscopy in STZ treated transgenic islet. Scale bar: 50 µm.
(0.27 MB TIF)

Figure S4 Real-time PCR result confirmed that the ngn3 transcript increased more than 300 times in dox and STZ treated transgenic mice, the values were normalized to control mice without STZ treatment. *P<0.05, n≥6
(0.20 MB TIF)

Figure S5 Islet cells in control mice after STZ treatment (A–C) Representative confocal images of co-staining with two endocrine markers. Dapi, Blue. (A) Denotes islets co-stained by Insulin (green) and Glucagon (red). (B) Denotes cells co-stained by Insulin (green) and Somatostain (red). (C) Denotes cells co-stained by Glucagon (red) and Somatostain (green). Scale bar: 20 µm.
(0.59 MB TIF)

Table S1 Primer sequences in RT-PCR.
(0.04 MB DOC)

## References

[1] Weir GC, Bonner-Weir S (2004). Five stages of evolving beta-cell dysfunction during progression to diabetes.. Diabetes.

[2] Shapiro AM, Lakey JR, Ryan EA, Korbutt GS, Toth E (2000). Islet transplantation in seven patients with type 1 diabetes mellitus using a glucocorticoid-free immunosuppressive regimen.. N Engl J Med.

[3] Robertson RP (2004). Islet transplantation as a treatment for diabetes - a work in progress.. N Engl J Med.

[4] Trucco M (2005). Regeneration of the pancreatic beta cell.. J Clin Invest.

[5] Like AA, Rossini AA (1976). Streptozotocin-induced pancreatic insulitis: new model of diabetes mellitus.. Science.

[6] Bonner-Weir S, Baxter LA, Schuppin GT, Smith FE (1993). A second pathway for regeneration of adult exocrine and endocrine pancreas. A possible recapitulation of embryonic development.. Diabetes.

[7] Rosenberg L (1998). Induction of islet cell neogenesis in the adult pancreas: the partial duct obstruction model.. Microsc Res Tech.

[8] Sandgren EP, Luetteke NC, Palmiter RD, Brinster RL, Lee DC (1990). Overexpression of TGF alpha in transgenic mice: induction of epithelial hyperplasia, pancreatic metaplasia, and carcinoma of the breast.. Cell.

[9] Nir T, Melton DA, Dor Y (2007). Recovery from diabetes in mice by beta cell regeneration.. J Clin Invest.

[10] Laybutt DR, Weir GC, Kaneto H, Lebet J, Palmiter RD (2002). Overexpression of c-Myc in beta-cells of transgenic mice causes proliferation and apoptosis, downregulation of insulin gene expression, and diabetes.. Diabetes.

[11] Dor Y, Brown J, Martinez OI, Melton DA (2004). Adult pancreatic beta-cells are formed by self-duplication rather than stem-cell differentiation.. Nature.

[12] Brennand K, Huangfu D, Melton D (2007). All beta Cells Contribute Equally to Islet Growth and Maintenance.. PLoS Biol.

[13] Xu X, D'Hoker J, Stange G, Bonne S, De Leu N (2008). Beta cells can be generated from endogenous progenitors in injured adult mouse pancreas.. Cell.

[14] Finegood DT, Scaglia L, Bonner-Weir S (1995). Dynamics of beta-cell mass in the growing rat pancreas. Estimation with a simple mathematical model.. Diabetes.

[15] Lee CS, De Leon DD, Kaestner KH, Stoffers DA (2006). Regeneration of pancreatic islets after partial pancreatectomy in mice does not involve the reactivation of neurogenin-3.. Diabetes.

[16] Teta M, Rankin MM, Long SY, Stein GM, Kushner JA (2007). Growth and regeneration of adult beta cells does not involve specialized progenitors.. Dev Cell.

[17] Bonner-Weir S, Trent DF, Honey RN, Weir GC (1981). Responses of neonatal rat islets to streptozotocin: limited B-cell regeneration and hyperglycemia.. Diabetes.

[18] Riley WJ, McConnell TJ, Maclaren NK, McLaughlin JV, Taylor G (1981). The diabetogenic effects of streptozotocin in mice are prolonged and inversely related to age.. Diabetes.

[19] Fernandes A, King LC, Guz Y, Stein R, Wright CV (1997). Differentiation of new insulin-producing cells is induced by injury in adult pancreatic islets.. Endocrinology.

[20] Guz Y, Nasir I, Teitelman G (2001). Regeneration of pancreatic beta cells from intra-islet precursor cells in an experimental model of diabetes.. Endocrinology.

[21] Suzuki A, Nakauchi H, Taniguchi H (2004). Prospective isolation of multipotent pancreatic progenitors using flow-cytometric cell sorting.. Diabetes.

[22] Sugiyama T, Rodriguez RT, McLean GW, Kim SK (2007). Conserved markers of fetal pancreatic epithelium permit prospective isolation of islet progenitor cells by FACS.. Proc Natl Acad Sci U S A.

[23] Kodama S, Toyonaga T, Kondo T, Matsumoto K, Tsuruzoe K (2005). Enhanced expression of PDX-1 and Ngn3 by exendin-4 during beta cell regeneration in STZ-treated mice.. Biochem Biophys Res Commun.

[24] Cozar-Castellano I, Haught M, Stewart AF (2006). The cell cycle inhibitory protein p21cip is not essential for maintaining beta-cell cycle arrest or beta-cell function in vivo.. Diabetes.

[25] Cozar-Castellano I, Weinstock M, Haught M, Velazquez-Garcia S, Sipula D (2006). Evaluation of beta-cell replication in mice transgenic for hepatocyte growth factor and placental lactogen: comprehensive characterization of the G1/S regulatory proteins reveals unique involvement of p21cip.. Diabetes.

[26] Wu H, Wade M, Krall L, Grisham J, Xiong Y (1996). Targeted in vivo expression of the cyclin-dependent kinase inhibitor p21 halts hepatocyte cell-cycle progression, postnatal liver development and regeneration.. Genes Dev.

[27] Whitson JM, Noonan EJ, Pookot D, Place RF, Dahiya R (2009). Double stranded-RNA-mediated activation of P21 gene induced apoptosis and cell cycle arrest in renal cell carcinoma.. Int J Cancer.

[28] Hume WJ, Potten CS (1979). Advances in epithelial kinetics–an oral view.. J Oral Pathol.

[29] Ohlsson H, Karlsson K, Edlund T (1993). IPF1, a homeodomain-containing transactivator of the insulin gene.. EMBO J.

[30] Beattie GM, Itkin-Ansari P, Cirulli V, Leibowitz G, Lopez AD (1999). Sustained proliferation of PDX-1+ cells derived from human islets.. Diabetes.

[31] Peshavaria M, Larmie BL, Lausier J, Satish B, Habibovic A (2006). Regulation of pancreatic beta-cell regeneration in the normoglycemic 60% partial-pancreatectomy mouse.. Diabetes.

[32] Gu G, Dubauskaite J, Melton DA (2002). Direct evidence for the pancreatic lineage: NGN3+ cells are islet progenitors and are distinct from duct progenitors.. Development.

[33] Alpert S, Hanahan D, Teitelman G (1988). Hybrid insulin genes reveal a developmental lineage for pancreatic endocrine cells and imply a relationship with neurons.. Cell.

[34] Polak M, Bouchareb-Banaei L, Scharfmann R, Czernichow P (2000). Early pattern of differentiation in the human pancreas.. Diabetes.

[35] Cano DA, Rulifson IC, Heiser PW, Swigart LB, Pelengaris S (2008). Regulated beta-cell regeneration in the adult mouse pancreas.. Diabetes.

[36] Garcia-Ocana A, Vasavada RC, Cebrian A, Reddy V, Takane KK (2001). Transgenic overexpression of hepatocyte growth factor in the beta-cell markedly improves islet function and islet transplant outcomes in mice.. Diabetes.

