# Supplementary figures and images for: P21 cip-Overexpression in the Mouse β Cells Leads to the Improved Recovery from Streptozotocin-Induced Diabetes
Source: PLoS One. 2009 Dec 17;4(12):e8344. doi: 10.1371/journal.pone.0008344 (PMC2792146; doi:10.1371/journal.pone.0008344)

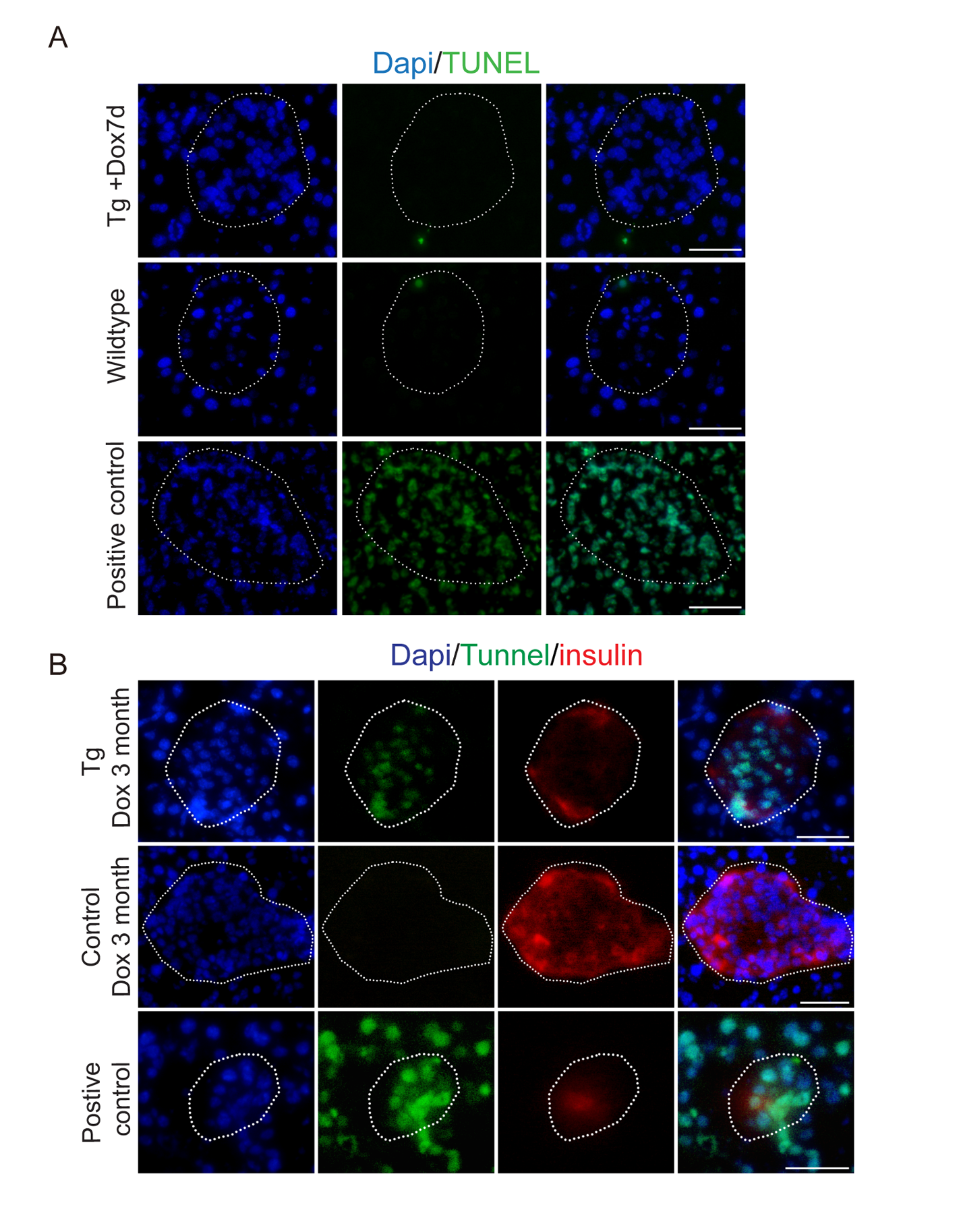

Supplement: Figure S1 — P21 over-expression caused apoptosis in double transgenic mice after dox treatment up to 3 months. Six-week-old mice were treated with doxycycline for one week (A) and 3 months (B), then sacrificed and assessed for apoptotic cell death. On 7th day, little β cell apoptosis was detected by TUNEL staining in double-transgenic mice as that in wildtype mice. After 3 months, β cell apoptosis was detected only in transgenic mice. Pancreatic slides treated with DNase I prior to the addition of the 3′-OH labeling mixture were utilized as positive control for this experiment. Islets were indicated by dashed circle. Scale bar: 50 µm. (2.21 MB TIF) [file pone.0008344.s001.tif]

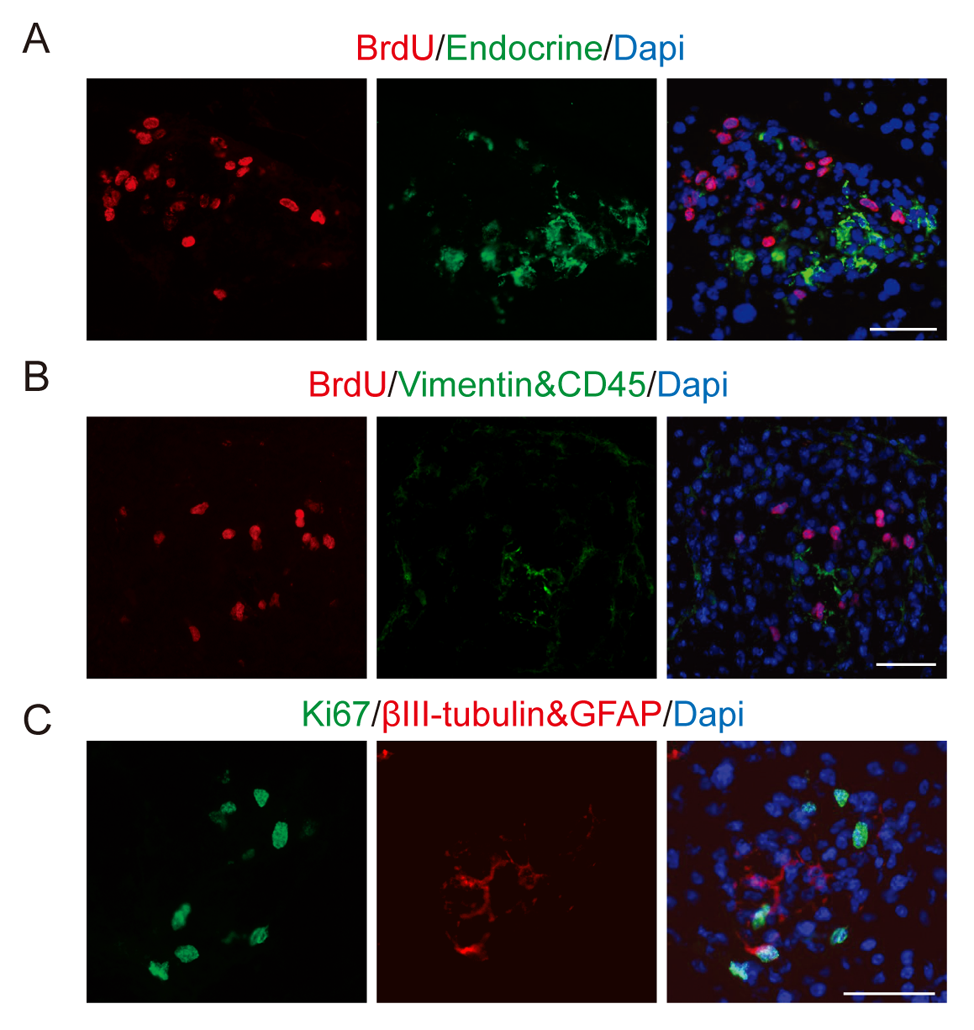

Supplement: Figure S2 — Proliferating cell did not co-stain with markers of terminally differentiated cells in islet. (A) Double-labeling with BrdU (red) and endocrine markers (green) such as Insulin, Glucagon, Somatostatin, and pancreatic polypeptide demonstrated that these proliferating cells are not endocrine cells - α, β, δ or pancreatic polypeptide cells. (B) Staining with Ki67 (green) and βIII-tubulin and GFAP (red) revealed that these proliferating cells are not neurons or astrocytes. (C) Staining with BrdU (red) and vimentin and CD45 revealed that these proliferating cells are not hematopoietic cells or mesenchymal cells. Scale bar, 50 µm. (0.79 MB TIF) [file pone.0008344.s002.tif]

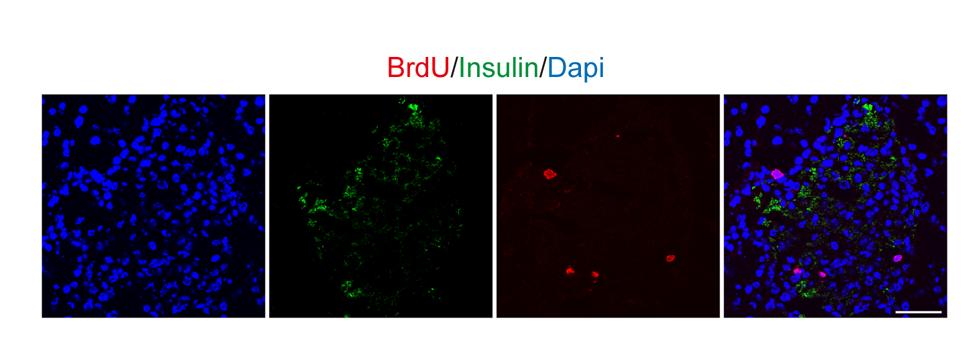

Supplement: Figure S3 — Proliferating cell did not co-stain with insulin in islet. Six-week-old mice were treated with dox for 7 days, and then injected with 200 mg/kg STZ. Two days after STZ treatment, Double-labeling with BrdU (red) and insulin (green) was checked by confocal microscopy in STZ treated transgenic islet. Scale bar: 50 µm. (0.27 MB TIF) [file pone.0008344.s003.tif]

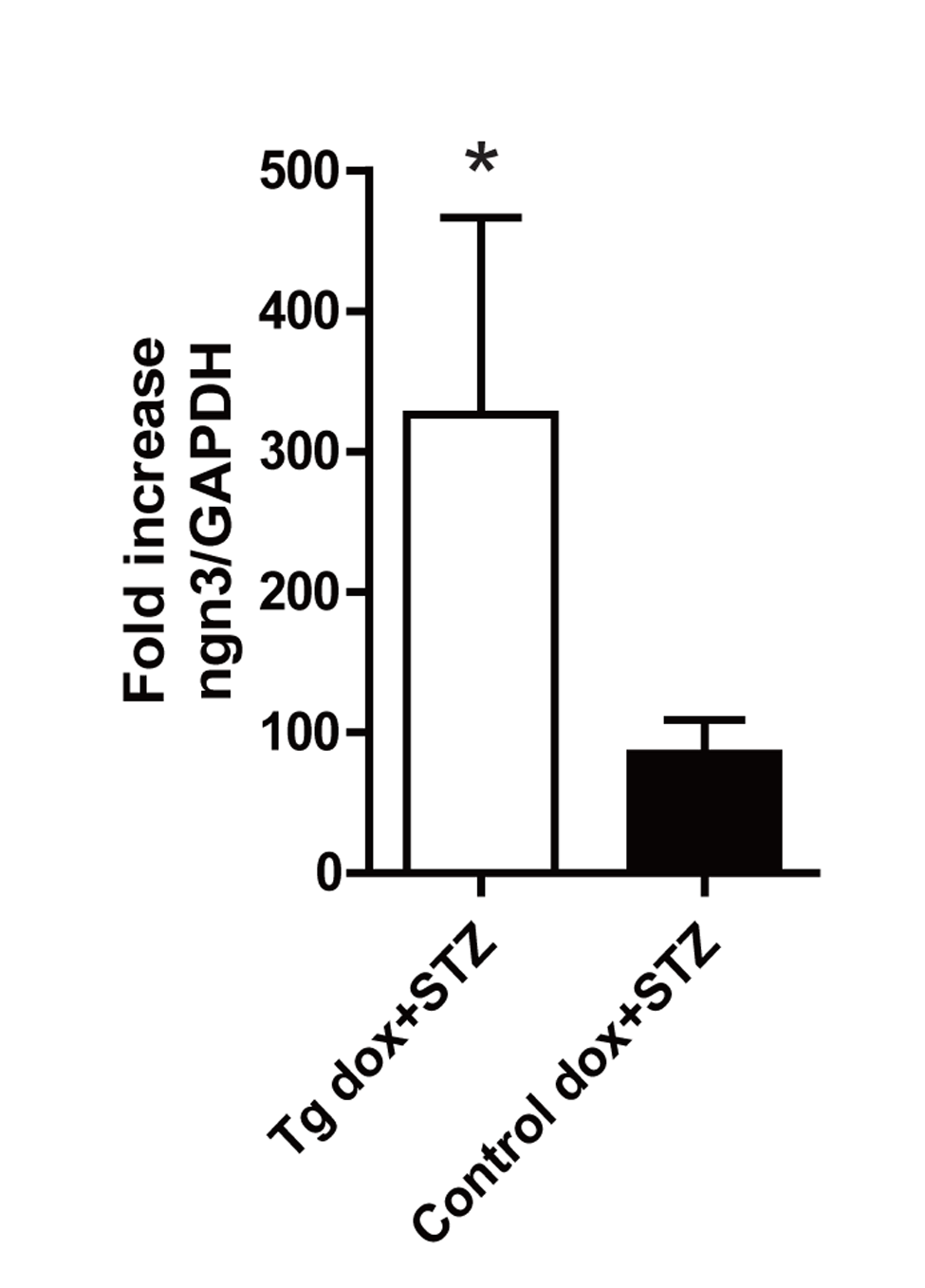

Supplement: Figure S4 — Real-time PCR result confirmed that the ngn3 transcript increased more than 300 times in dox and STZ treated transgenic mice, the values were normalized to control mice without STZ treatment. *P<0.05, n≥6 (0.20 MB TIF) [file pone.0008344.s004.tif]

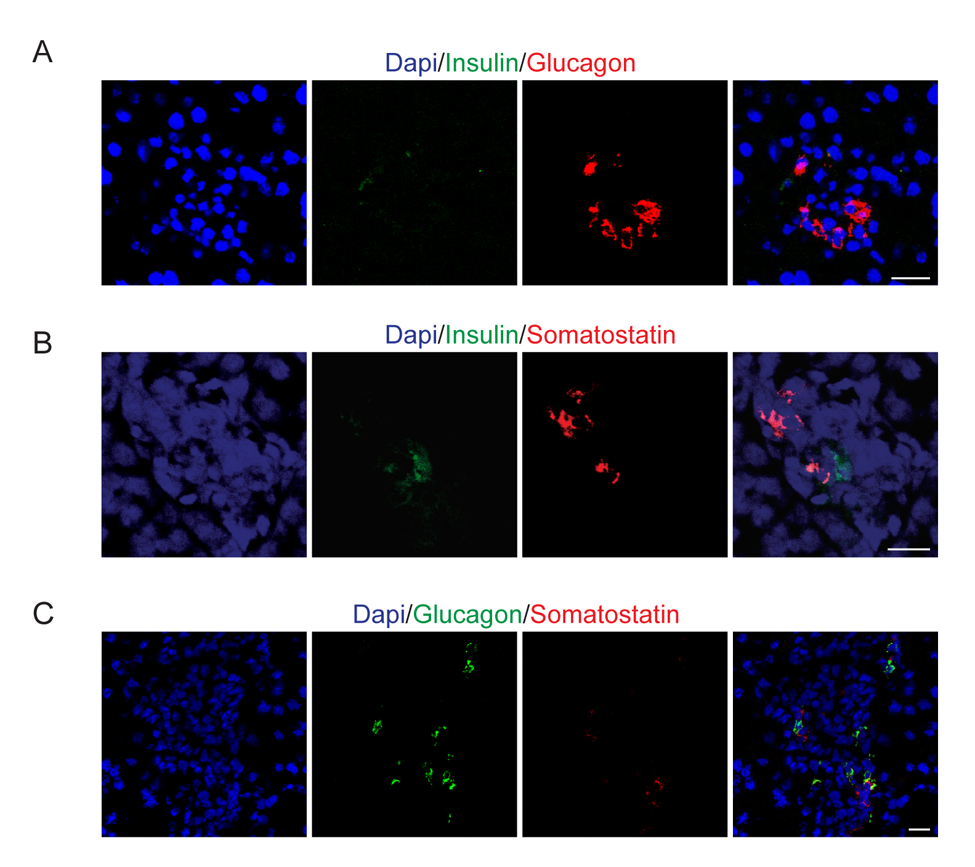

Supplement: Figure S5 — Islet cells in control mice after STZ treatment (A–C) Representative confocal images of co-staining with two endocrine markers. Dapi, Blue. (A) Denotes islets co-stained by Insulin (green) and Glucagon (red). (B) Denotes cells co-stained by Insulin (green) and Somatostain (red). (C) Denotes cells co-stained by Glucagon (red) and Somatostain (green). Scale bar: 20 µm. (0.59 MB TIF) [file pone.0008344.s005.tif]
