# Supplementary material for: P21 cip-Overexpression in the Mouse β Cells Leads to the Improved Recovery from Streptozotocin-Induced Diabetes
Source: PLoS One. 2009 Dec 17;4(12):e8344. doi: 10.1371/journal.pone.0008344 (PMC2792146; doi:10.1371/journal.pone.0008344)
Supplement: Table S1 — Primer sequences in RT-PCR. (0.04 MB DOC) [file pone.0008344.s006.doc]

**Table S1** Primer sequences in RT-PCR

| Gene name | Forward (5’-3’) | Reverse (5’-3’) |
| --- | --- | --- |
| *Pdx1* | AGGTCACCGCACAATCTTGCT | CTTTCCCGAATGGAACCGA |
| *Ngn3* | CAGTCACCCACTTCTGCTTC | GAGTCGGGAGAACTAGGATG |
| *Hnf6* | GCAATGGAAGTAATTCAGGGCAG | CATGAAGAAGTTGCTGACAGTGC |
| *Ptf1α* | AACCAGGCCCAGAAGGTTAT | CCTCTGGGGTCCACACTTTA |
| *Sox9* | GGCGGAGGAAGTCGGTGAAG | GGGTGCGGTGCTGCTGAT |
| *Nkx2.2* | ACTTGGACGGGAACTGACAC | CTAAATATTTATGGCCATGTACACG |
| *Pax6* | AACAACCTGCCTATGCAACC | ACTTGGACGGGAACTGACAC |
| *IA1* | CGGGCGCTGCTGCTGTCAC | CCGGCGAGCCCAGGTTGAAG |
| *NeuroD1* | GTCCCAGCCCACTACCAATT | CGGCACCGGAAGAGAAGATT |
| *Pax4* | TGAAGTGCCCGAAGTACTCGA | AGGCAGCAGATTGTGCAGCTA |
| *Arx* | AGTGGCGCAAGCGGGAGAAG | GGCGGGTGTGGGCTGTCT |
| *Nkx6.1* | CCGGTCGGACGCCCATC | GAGGCTGCCACCGCTCGATTT |
| *Glucagon* | CCACTCACAGGGCACATTCA | GTCCCTGGTGGCAAGATTGT |
| *Ptc1* | GTCTCAGGGTAGCTCTCATA | GCATTCTGGCCCTAGCAATA |
| *Gli1* | TGTGGCGAATAGACAGAGGT | TGCCAGATATGCTTCAGCCA |
| *Gli2* | CTGGCCATACTAGTATAGCG | TTCATGGAGTCCCAGCAGAA |
| *Gli3* | TGAAGCATCTGTGGAGATCC | AAGTGACCTGTCAGGTGTAG |
| *Notch1* | CGGTGAACAATGTGGATGCT | ACTTTGGCAGTCTCATAGCT |
| *Notch2* | GTGGAGGCGACTCTTCTGCT | GCTGGGAGTCACGTTATACT |
| *Notch3* | GAGGCTACCTTGGCTCTGCT | GGCAGCCTGTCCAAGTGATCT |
| *Dll4* | CTGTCCTTATGGCTTTGTGG | GCTCCTTCTTCTGGTTGTG |
| *Jag1* | CCAGCCAGTGAAGACCAAGT | TCAGCAGAGGAACCAGGAAA |
| *Gapdh* | CAACAGCCTCAACATCATCAG | GGTCCACCACTGACACGTTG |
